# Associations of hyponatremia and SIADH with increased mortality, young age and infection parameters in patients with tuberculosis

**DOI:** 10.1371/journal.pone.0275827

**Published:** 2022-10-13

**Authors:** Christina Bal, Daniela Gompelmann, Michael Krebs, Lukasz Antoniewicz, Claudia Guttmann-Ducke, Antje Lehmann, Christopher Oliver Milacek, Maximilian Robert Gysan, Peter Wolf, Maaia-Margo Jentus, Irene Steiner, Marco Idzko

**Affiliations:** 1 Department of Pneumology, University Hospital Vienna AKH, Medical University of Vienna, Vienna, Austria; 2 Department of Medicine III, Division of Endocrinology and Metabolism, University Hospital Vienna AKH, Medical University of Vienna, Vienna, Austria; 3 Center for Medical Statistics, Informatics, and Intelligent Systems (CeMSIIS), Section for Medical Statistics, Medical University of Vienna, Vienna, Austria; Shandong Public Health Clinical Center: Shandong Provincial Chest Hospital, CHINA

## Abstract

**Background and objective:**

Hyponatremia and the syndrome of inappropriate antidiuretic hormone secretion (SIADH) are associated with and can be caused by tuberculosis (TB) through meningitis by locally invading the hypothalamus, adrenal, or pituitary glands or possibly through ectopic ADH production. This study assessed the association of TB mortality with hyponatremia and SIADH in a large cohort of a university hospital in Austria.

**Methods:**

This retrospective study enrolled patients with hyponatremia and patients diagnosed with TB from 01/2001-11/2019 to assess the prevalence of TB in hyponatremia and TB morbidity and mortality in patients with and without hyponatremia. Sex, age, microbiological results, laboratory tests and comorbidities were analysed and used to calculate survival rates.

**Results:**

Of 107.532 patients with hyponatremia (0.07%) and 186 patients with TB (43%), 80 patients were diagnosed with both—hyponatremia and TB. Only three TB patients had SIADH, precluding further SIADH analysis. In hyponatremia, young age and high CRP levels showed significant associations with TB diagnosis (p<0.0001). Survival rates of patients diagnosed with TB with moderate to profound hyponatremia were significantly lower than those without hyponatremia (p = 0.002).

**Conclusion:**

In this study of a large cohort from a tertiary care hospital in a non-endemic area of TB, 0.07% of patients presenting with hyponatremia, but especially younger patients and patients with high CRP values, were diagnosed with TB. Crucially, patients with moderate to profound hyponatremia had a significantly higher mortality rate and thus required increased medical care.

## Introduction

Hyponatremia and the syndrome of inappropriate antidiuretic hormone secretion (SIADH) are associated with [1–3] and can be caused [4] by the infectious disease tuberculosis (TB). SIADH causes about a third of all hyponatremia episodes [5]. It is a hypotonic euvolemic hyponatremia subform with urine osmolality exceeding >100 mOsmol/kg and sodium content >30 mEq/L, absent of any other causative hormonal issues or Antidiuretic Hormone (ADH) suppression [1, 6]. Hyponatremia also arises from numerous other causes, including renal failure, cerebral salt-wasting syndrome, diuretic therapy, and other medications [1]. Assessing volume and urine status adequately and excluding renal, hepatic, cardiac, and other endocrinal causes for SIADH diagnosis to distinguish the correct hyponatremia cause is essential for treatment. In a systematic review, patients with TB showed generally adequate renal and adrenal functionality, affirming SIADH as a frequent underlying mechanism of hyponatremia in TB [7]. TB was reported to cause SIADH by displacing relevant functional cells through invasion of the hypothalamus, the adrenal or pituitary gland in TB meningitis, or possibly by route of pulmonary infections with ectopic ADH production [2, 8]. A critical unknown is the primary pathophysiological pathway through which TB causes SIADH: the first demonstration of ADH activity caused by TB in active inflammatory lung tissue vs regular tissue was already demonstrated in 1970 [9], yet current progress in the pathophysiology could only reassess this hypothesis in a case report [10]. In fact, pituitary hormone analysis was only performed in a single study of TB meningitis [4], and no study to date differentiates ectopic vs pituitary deranged ADH production in TB patients [8]. Mice models show that genes associated with ADH production are upregulated from early infection and involve macrophages as a principal target, possibly causing increased disease burden [11]. Further elucidation of TB-associated SIADH in pathophysiological studies would shed light on this clinically relevant effect.

Hyponatremia itself leads to various symptoms, from subclinical to severe, and is consistently associated with worse outcomes and increased mortality [2, 12].

However, few studies analysed the effects of hyponatremia in TB [13], especially regarding episodes of SIADH. Furthermore, prior studies either had a high disparity in patient population groups regarding severity, TB location, and patient characteristics or did not survey patients with proven TB diagnosis [3, 14].

The primary objective of this paper was to assess the prevalence, and association of TB morbidity and mortality with hyponatremia and SIADH in a large cohort of a university hospital, to better provide information for more informed choices in monitoring and care of patients with TB in non-endemic areas.

## Methods

### Patient recruitment

**Fig 1:**
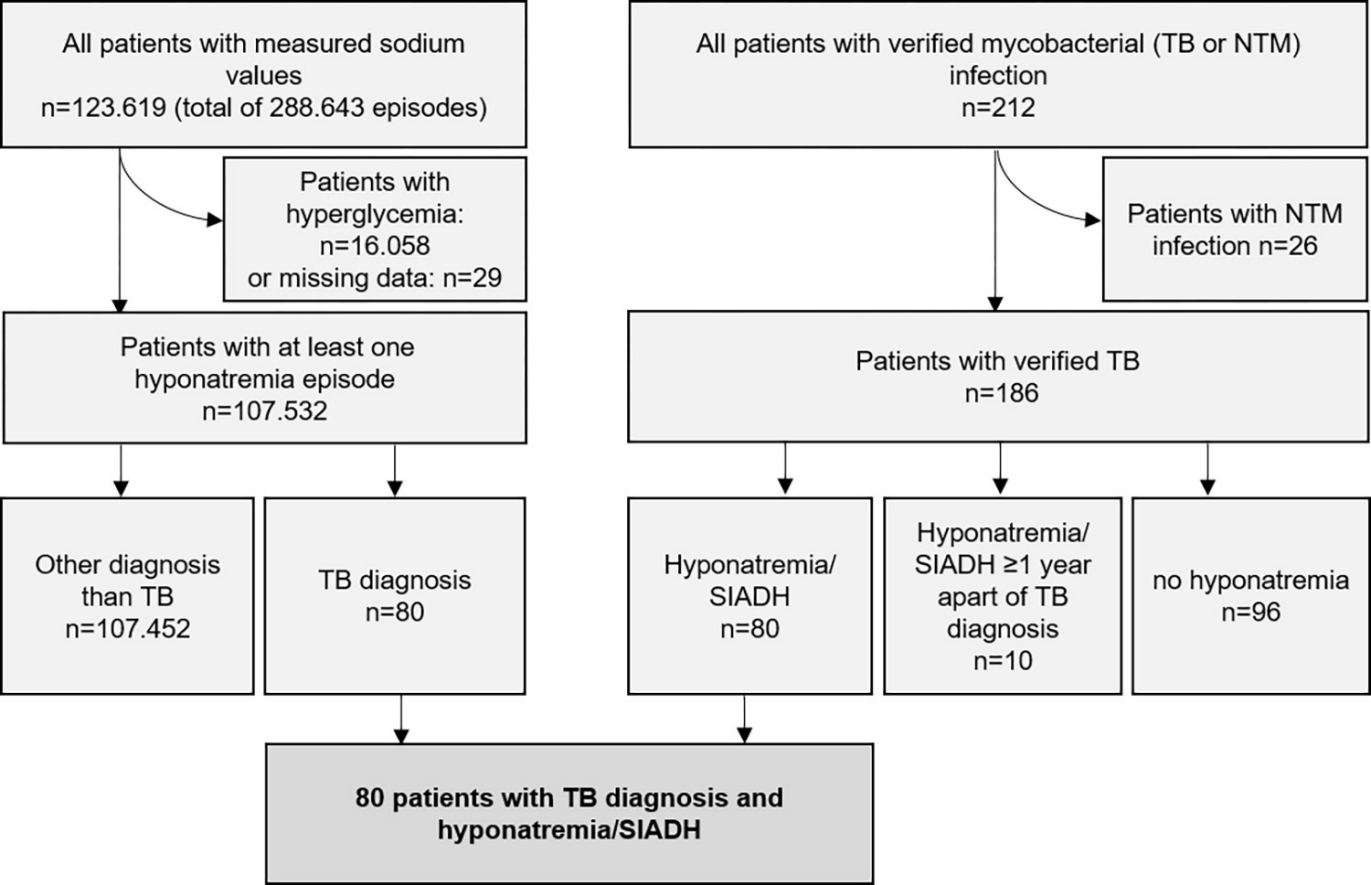
Study cohort. Same-day serum osmolarity <275 mOsm/kg, urine osmolarity >100 mOsm/kg, and urine sodium excretion >30 mmol/L identified SIADH (1087 of 7055 patients tested satisfied all criteria). Hyponatremia, as noted in the flowchart, excluded hyperglycemia. TB, tuberculosis. SIADH, syndrome of inappropriate antidiuretic hormone secretion.

We acquired patient data retrospectively from the tertiary hospital of the medical university of Vienna from 01/2001-11/2019 (Fig 1). All data was sourced from medical records and de-identified. Patients were included in the study if TB or hyponatremia was recorded, matching the parameters below. This observational study was performed in accordance with the Declaration of Helsinki and approved by the Ethics Committee of the Medical University of Vienna. The ethics committee waived the need for consent because of the study’s retrospective nature.

### Parameters

Hyponatremia episodes began at the first measurement of sodium level of <136 mmol/L in serum (S1 Table). SIADH applied to hyponatremia episodes if the definition of Schwartz and Bartter [6], in agreement with current guidelines [1, 15], was fulfilled: same-day serum osmolarity <275 mOsm/kg, urine osmolarity >100 mOsm/kg and urine sodium excretion >30 mmol/L. A straightforward method to establish SIADH for this study was adopted from Hoorn et al. 2017, as it provided a consistent urine sodium excretion cut-off (≥ 30 mmol/L) which could exclude with acceptable sensitivity and specificity heart failure and liver cirrhosis as well as nephrotic syndrome. Urine sodium excretion also indirectly identified euvolemia [15], particularly as no manual patient record assessment for SIADH for such an extensive database was possible. However, renal and cerebral salt wasting and rare or multi-faceted causes of hyponatremia are not integrated well enough in this concise approach and necessitate exclusion on a case-by-case basis in clinical praxis.

Two consecutive measurements of sodium levels in serum >135 mmol/L or a maximum of 30 days, to account for out-patient measurements [16], defined the end of an episode. The lowest serum sodium value of an episode assigned hyponatremia severity as mild (135–130 mmol/L), moderate (129–125 mmol/L) or profound (<125 mmol/L) [1].

The first positive Mycobacterium tuberculosis culture, Polymerase chain reaction (PCR) result or Ziehl-Neelsen stain defined TB diagnosis (S2 Table). Subsequent positive TB tests or TB episodes in the same patient were excluded from the analysis. Infections of the central nervous system (CNS), miliar TB, or pulmonary TB marked different localisations. Out-patient, in-patient or intensive care treatment modality distinguished TB severity. The analysis excluded patients with only clinical suspicion of TB but included all verified TB cases.

Patients with TB were assigned to groups according to hyponatremia severity if at least one hyponatremia episode occurred within one year of TB diagnosis and to groups without hyponatremia if no hyponatremia or an episode more than one year apart from TB diagnosis arose. This method enabled a comprehensive detection of patients with both TB and hyponatremia [17].

The analysis included peak C-Reactive Protein (CRP, mg/dl) and leucocyte levels (G/L) as continuous variables to mark inflammation status within each episode. A CRP level of >0.5 mg/dl and a leucocyte level >7.5 G/L were considered elevated. An assessment of overall survival covered the interval between TB diagnosis and death using the date last seen or death recorded in the Austrian national death registry.

### Statistical analysis

If not stated otherwise, we reported quantitative variables as mean ± standard deviation (SD) and qualitative variables as absolute frequencies and percentages. Estimates with 95% Clopper-Pearson confidence intervals delineate the prevalence of TB. To calculate the effect of age, sex and infection parameters (CRP, leucocyte levels) on the development of TB in hyponatremia patients, we performed univariate logistic regression models for correlated data (SAS Proc genmod), whereby an independent correlation structure was assumed.

If the univariate model revealed a p-value less than 0.05, the variable entered a multivariable logistic regression model for correlated data. The odds ratio (OR) with 95% confidence limits (CI) and the p-value (H0: OR = 1) report the results of each model. We excluded from analysis all episodes starting after TB diagnosis (n = 159 episodes of 56 patients) because of possible treatment interference.

We performed a Kaplan-Meier curve including all 186 patients with TB to analyse the overall survival in patients in groups separated by hyponatremia at 1, 5, and 10 years (reported with 95%CI; until query 10/2019). We analysed the effect of hyponatremia on overall survival through a Cox regression adjusting for age and comorbidities (R-package survival, R-function coxph). All evaluations were performed with R 3.6.1 and SAS 9.4. P values <0.05 defined statistical significance. Reports followed Strengthening the Reporting of Observational studies in Epidemiology (STROBE) guidelines [18] (S1 Appendix).

## Results

**Fig 2:**
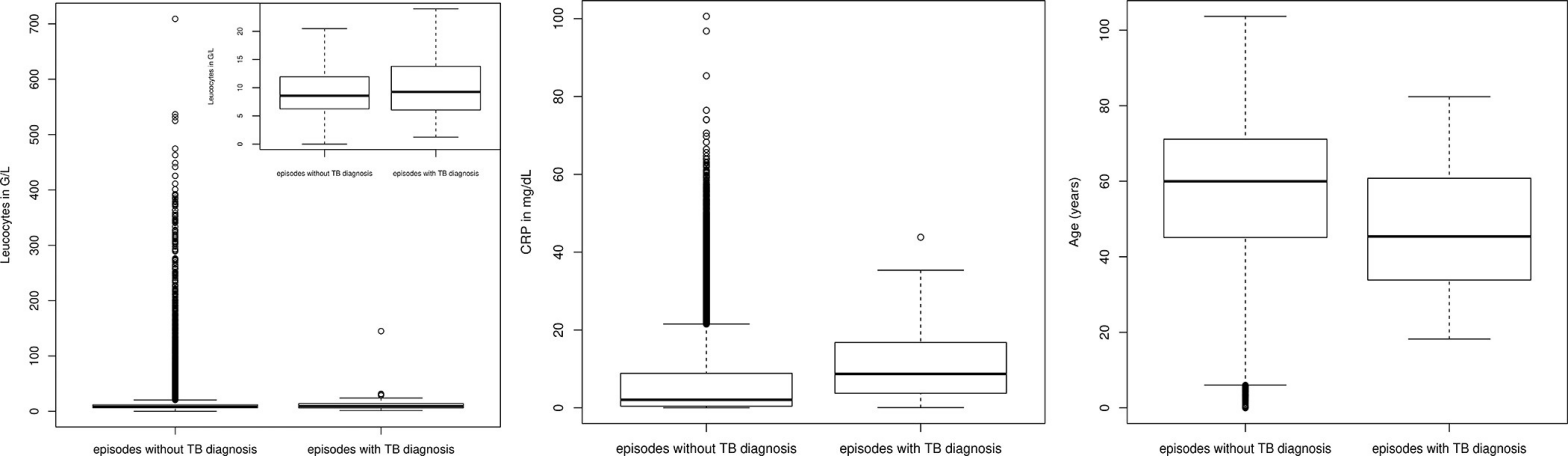
Infection parameters and age in patients with hyponatremia. Patients with TB showed higher leucocyte count (A), higher CRP values (B) and younger age (C) than patients without TB. Boxplots mark first IQR, median, and third IQR. The Whiskers extend to the minimum and maximum, and if outliers exist (shown as open circles), to the smallest and largest value within the interval [first quartile –1.5x IQR; third quartile +1.5x IQR].

The analysis included 107.532 patients with at least one hyponatremia episode (mean age 58 ± 18 years, 52% female) with a total of 272.579 hyponatremia episodes and 186 patients with TB irrespective of hyponatremia. Eighty patients (0.07% [95%CI: 0.06%; 0.09%]) had both TB and hyponatremia, with a mean of 2.64 hyponatremia episodes within the designated timeframe (Figs 1 and 2).

Of 212 patients diagnosed with mycobacterial infections, 186 had tuberculosis (mean age 44 ± 19 years, 39% female) and 26 non-tuberculosis mycobacteria (NTM). Out of the 186 patients with TB, 80 patients (43.0%) developed hyponatremia within a year of TB diagnosis, whereby laboratory tests revealed mild, moderate or profound hyponatremia in 62 (77.5%), 13 (16.3%) and 5 (6.3%) patients respectively. Ten patients (5.4%) presented hyponatremia more than one year apart from the TB diagnosis, whereas 96 patients (51.6%) had no hyponatremia episodes within the observation period.

Of 7055 patients tested for SIADH, 1087 satisfied all criteria. Only three patients (0.28% [95%CI: 0.06%; 0.80%]) with verified SIADH were diagnosed with TB (Table 1). The small patient number precluded further analysis.

**Table 1:** Characteristics of TB in patients grouped by hyponatremia. Patients with TB and hyponatremia had more frequent infectious, pulmonary, malignant and cardiovascular comorbidities. When we analysed the cardiovascular comorbidities in-depth, neither arterial hypertonia nor coronary heart disease was associated with increased hyponatremia. TB severity was not associated with hyponatremia. Group comparison is shown with absolute frequencies (n), percentages with 95% CI, and unadjusted p-values (Chi-squared tests, if not stated otherwise). Note that the interpretation of the p-values is descriptive. TB, tuberculosis. CNS, central nervous system. COPD, chronic obstructive pulmonary disease. HIV, human immunodeficiency virus infection. ICU, intensive care unit. GCS, Glasgow Coma Scale.

	hyponatremia (n = 80)			no hyponatremia (n = 106)			
TB location	n	% of total	95% CI	n	% of total	95% CI	p-value
at least pulmonary TB	50	63%	[51; 73]	54	51%	[41; 61]	0.2
isolated pulmonary TB	31	39%	[28; 50]	37	35%	[26; 45]	0.7
CNS–TB	3	4%	[0.8; 11]	2	2%	[0.2; 7]	0.7^1^
TB in any other organ	34	43%	[32; 54]	32	30%	[22; 40]	0.1
Comorbidity							
Cardiovascular comorb.	22	28%	[18; 39]	17	16%	[10; 24]	0.09
• arterial hypertonia	13	16%	[9; 26]	10	9%	[5; 17]	0.2
• coronary heart disease	6	8%	[3; 16]	8	8%	[3; 14]	1
Diabetes mellitus	8	10%	[4; 19]	13	12%	[7; 20]	0.8
Endocrine	24	30%	[20; 41]	24	23%	[15; 32]	0.3
Gastrointestinal	10	13%	[6; 22]	7	7%	[3; 13]	0.3
Immunosuppress. therapy	15	19%	[11; 29]	10	9%	[5; 17]	0.1
Infectious disease, any*	38	48%	[36; 59]	23	22%	[14; 31]	<0.001
• HIV	13	17%	[9; 26]	3	3%	[0.6; 8]	0.003
• Hepatitis C	6	8%	[3; 16]	2	2%	[0.2; 6]	0.08^1^
Neurological	18	23%	[14; 33]	12	11%	[6; 19]	0.06
Oncological	18	23%	[14; 33]	9	8%	[4; 16]	0.013
Pulmonary comordbidity	25	31%	[21; 43]	18	17%	[10; 26]	0.035
• COPD	8	10%	[4; 19]	6	6%	[2; 12]	0.4
Renal	15	19%	[11; 29]	11	10%	[5; 18]	0.2
TB severity							
Mild (Outpatient)	11	14%		21	20%		0.3^2^
Moderate (Inpatient)	67	84%		82	77%		
• Moderate, with neurologic symptoms	15	19%		5	5%		
Severe (ICU)	2	3%		3	3%		
• Severe, with GCS 3	2	3%		0	0%		

*: including Hepatitis A, B, C, D; HIV, aspergillosis, genital herpes, abscess, pneumocystis, erysipelas, toxoplasmosis, and urinary tract infection.

^1^Fisher’s exact test

^2^Wilcoxon rank-sum test.

### Predictors for TB diagnosis in patients with hyponatremia

**Fig 3:**
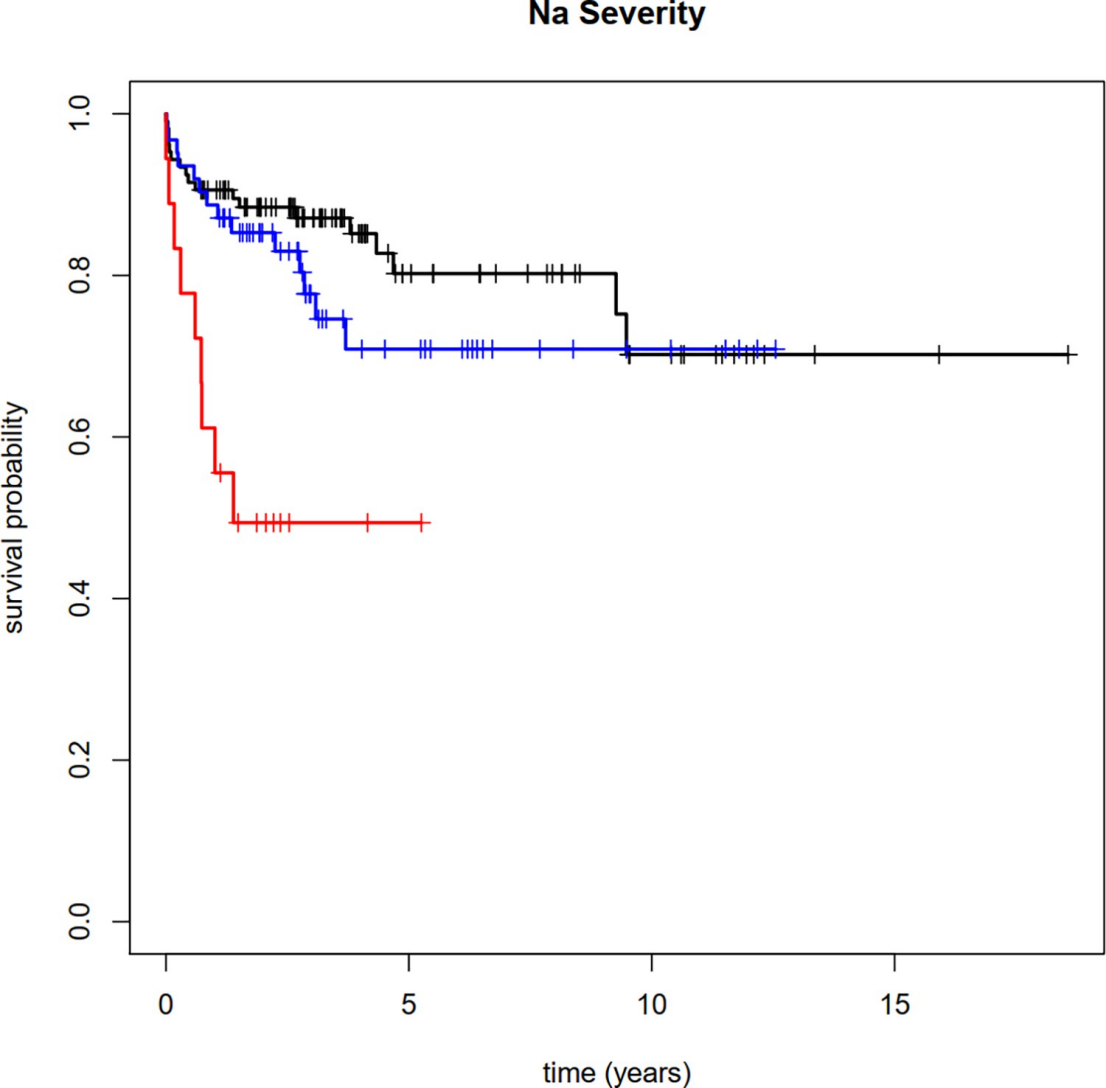
Mortality risk in patients with TB. The graph details the mortality rates of patients with TB with moderate to profound hyponatremia (red), mild hyponatremia (blue), and non-hyponatremic patients (black).

In univariate logistic regression models, young age and elevated infection parameter levels showed associations with an increased risk of TB diagnosis. Every CRP level increase by 1 mg/dl increased the odds [95%CI] of TB infection by 1.053 [1.038; 1.068] (p<0.0001), and leucocyte count increase of 1 G/L by 1.006 [1.005; 1.0115] (p = 0.032). Sex was not significantly associated with TB diagnosis (p = 0.8). In a multivariable logistic regression model, the influence of age and CRP levels remained statistically significant (p<0.0001, Fig 3 and Table 2).

**Table 2:** Epidemiologic factors associated with TB diagnosis in patients with hyponatremia. Young age and high CRP parameters conferred a higher TB diagnosis risk in patients with hyponatremia in a multiple logistic regression model for correlated data. TB, tuberculosis. OR, odds ratio. CRP, C-reactive protein. n, number of patients.

parameter	univariate models			n	multiple model			n
	OR	95% CI	p-value		OR	95% CI	p-value	
sex	0.92	0.50, 1.71	0.8	272309 (107465)	-	-	-	259641 (103972)
age	0.97	0.95, 0.98	<0.0001	272309 (107465)	0.97	0.95, 0.98	<0.0001	
CRP	1.05	1.04, 1.07	<0.0001	264054 (105103)	1.05	1.04, 1.07	<0.0001	
leucocytes	1.01	1.01, 1.01	0.032	264023 (105019)	1.00	0.97, 1.03	0.9	

### Survival analysis

Of 186 patients diagnosed with TB, 80 had ≥1 hyponatremia episode within a year of TB diagnosis, including 62 patients with mild hyponatremia and 18 with moderate to profound hyponatremia; 96 had no hyponatremia within the observation period. Additionally, ten patients had episodes which did not overlap with the diagnosis.

In patients with moderate to profound hyponatremia, the proportion of patients that survived was 61.1% [42.3%; 88.3%] after 1 year and 49.4% [30.8%; 79.3%] after 5 years with no observational follow-up data available for the time point of 10 years. In patients with mild hyponatremia, the proportion of patients that survived was 88.7% [81.2%; 96.9%] after 1 year, 70.9% [58.2%; 86.2%] after 5 years, and 70.9% [58.2%; 86.2%] after 10 years. Of 106 patients without related hyponatremia, the proportion of patients that survived was 90.6% [85.2%; 96.3%] after 1 year, 80.2% [71.1%; 90.5%] after 5 years and 70.2% [56.3%; 87.6%] after 10 years.

After adjusting for age and comorbidity, mortality was significantly higher in patients with moderate or profound hyponatremia compared to patients without hyponatremia (HR [95%CI]: 3.7 [1.6; 8.6], p = 0.002) (Table 3).

**Table 3:** Increased mortality in patients with TB with moderate to profound hyponatremia. Patients with TB and moderate to profound hyponatremia episodes had a significantly higher all-cause mortality rate. After correcting for hyponatremia-related comorbidities (Table 1), the effect remains significant. A Cox regression model was calculated with hyponatremia groups and age as independent variables and reported as hazard ratio (HR) with a 95% confidence interval (95%LL, 95% UL) and p-value (H0: HR = 1). TB, tuberculosis. LL, lower limit. UL, upper limit. Q1, first quartile. Q3, third quartile.

a. Characterisation of hyponatremia profundity in patients with TB						
Hyponatremia severity	n	min	Q1	median	Q3	max
Non-hyponatremic (serum sodium ≥136 mmol/L)	106	136	-	-	-	-
Mild hyponatremia	62	130	132	134	135	135
Moderate hyponatremia	13	125	127	128	129	129
Profound hyponatremia	5	116	119	121	121	124
b. Parameters associated with mortality in TB patients—univariate model						
Parameter	HR	95% LL	95% UL	p-value	logrank test	
Mild hyponatremia (serum sodium 130–135 mmol/L) vs. non-hyponatremic	1.43	0.71	2.88	0.3	<0.0001	
Moderate and profound hyponatremia (serum sodium <130 mmol/L) vs. non-hyponatremic	4.87	2.14	11.06	0.0002		
Age (years)	1.06	1.04	1.08	<0.0001		
c. Parameters associated with mortality in TB patients—multivariable model						
Parameter	HR	95% LL	95% UL	p-value	type 3 test	
Mild hyponatremia (serum sodium 130–135 mmol/L) vs. non-hyponatremic	1.15	0.57	2.32	0.7	0.007	
Moderate and profound hyponatremia (serum sodium <130 mmol/L) vs. non-hyponatremic	3.7	1.59	8.64	0.002		
Age (years)	1.06	1.04	1.08	<0.0001		
d. Parameters associated with mortality in TB patients—multivariable model including adjustment for comorbidities						
Parameter	HR	95% LL	95% UL	p-value	type 3 test	
Mild hyponatremia (serum sodium 130–135 mmol/L) vs. non-hyponatremic	0.95	0.43	2.10	0.9	0.006	
Moderate and profound hyponatremia (serum sodium <130 mmol/L) vs. non-hyponatremic	3.68	1.53	8.83	0.004		
Age (years)	1.06	1.04	1.08	<0.0001		
Any pulmonary comorbidity	1.25	0.63	2.51	0.5		
Any infectious comorbidity	1.13	0.54	2.38	0.7		
Any oncological comorbidity	1.55	0.73	3.30	0.3		

None of the three patients with TB and SIADH died within the observation period. The death of five patients was directly attributed to TB; of these, all five had hyponatremia, and none had SIADH.

## Discussion

TB prevalence was rare and reflected a non-endemic area in this large cohort from a tertiary care hospital. Few patients presenting with hyponatremia were subsequently diagnosed with TB, and the diagnosis was mainly associated with young age and elevated infection parameters. In the current study, patients with TB and moderate to profound hyponatremia before or at diagnosis showed a significantly increased mortality rate.

This study of a low-incidence area [19] showed few TB diagnoses in patients with hyponatremia (0.07%) or SIADH (0.28%) in contrast to other studies regarding SIADH, where rates of TB-associated SIADH episodes ranged from 11% of initially idiopathic episodes [5] to 10% of all SIADH episodes plus further 3% of all idiopathic episodes after a one-year follow-up [17]. The highest previously reported prevalence reached up to 15 detections of TB in 46 intensive care patients with SIADH [2]. One primary reason for the discrepancy in rate may be the focus on idiopathic SIADH, which excluded other common causes. Hsu et al. further discussed that the results were probably due to a high local TB prevalence and a targetted prevention program with chest x-ray examinations [17, 20]. The small number of patients in the current study ultimately precluded an in-depth assessment of TB-associated SIADH. Conclusively, TB should be considered in unexplained causes of hyponatremia up to a year after the episode.

In the current study, younger age and higher CRP values conferred a higher likeliness of TB diagnosis in patients with hyponatremia, whereas other studies corroborated this effect of infection parameters [2, 21], indicating that signs of infection in this constellation potentially warrant a thorough screening for tuberculosis [21]. Likewise, our study reaffirmed other studies explaining that sex was irrelevant regarding TB in patients with hyponatremia [8, 17].

In terms of patients with TB, almost half of the patients in the current study had prior hyponatremia and 2% SIADH, comparable to other studies showing high prevalences of hyponatremia with up to 51%-60% presenting hyponatremia as the first symptom [2, 8]. In another study, 45% of patients with TB were similarly diagnosed with hyponatremia and 4% with SIADH [13]. A recent review summarised that hyponatremia was registered in more than half of TB cases, with older age being a risk factor [7, 8, 22, 23], corroborating the results of our study. We circumvented the age bias by performing an age-adjusted analysis as hyponatremia is known to be generally more frequent in older patients [1]. Hyponatremia was reportedly seen more often in patients who had lowered Glasgow Coma Scale scores and cerebral infarction [13, 24]. Neurologic comorbidities showed a trend towards more hyponatremia in the current study; however, neither neurologic TB localisation nor neurologic complications in our severity analysis yielded an association with hyponatremia, possibly because of a disparity in patient numbers. The analysis of different TB severity levels and locations in the current study, including CNS, miliar, and pulmonary TB, did not yield a significant difference in hyponatremia. However, the current study is highly underpowered for this analysis. Furthermore, our study only registered three patients with TB and SIADH. TB involving the CNS reportedly causes SIADH frequently, followed by pulmonary TB [2], with varied increased prevalences [3] of SIADH in TB meningitis ranging from 9.6% [4] to 50% [25] in all patients with TB and 39% in children [26], where it was associated with increased intracerebral pressure [26].

Further studies to evaluate morbidity and mortality in such patients are warranted. Clinically, a workup including blood cell count and CRP can direct the search for the cause of hyponatremia towards infections and lead to consideration of TB, whilst a higher disease burden warrants increased diagnostic efforts [21, 27].

Crucially, in the current study, patients with TB and episodes of moderate to profound hyponatremia prior to treatment had a significantly higher mortality rate than patients without hyponatremia. Even though TB is reportedly associated with hyponatremia and hyponatremia with increased mortality, different studies described their connection to the mortality rate differently, only some corroborating our results [3, 12, 26–28]. In one study of miliary TB, hyponatremia was one of two independent risk factors for requiring mechanical ventilation, with values above 124 mmol/L predicting a favourable outcome [12]. In children with TB, meningitis increased mortality (52%) vs those without hyponatremia (13%). As the study did not provide cases with proven TB infection, the statement could be of limited validity [14]. However, in other studies, only older age was a risk factor for mortality [13, 22], with possible reasons for the discrepancy including low TB severity in some cohorts, another that hyponatremia was mild or self-limited [8]. The current study included a large cohort of patients and was inclusive of all TB localisations [29] and comorbidities [26, 30, 31], and could detect an association between moderate to profound hyponatremia and increased mortality in patients with TB.

Evaluation of mortality and associated risk factors has become especially relevant in the advent of multidrug-resistant TB [32] and diversified TB therapy regimens [33]. Regarding patients with TB and SIADH in our study, only three patients with TB exhibited an episode of SIADH, and none died. Other studies underscored associations between SIADH and mortality, firstly in TB meningitis in multiple regression analysis [26], further in children with a mortality rate of 17% in SIADH vs 0% [27], lastly in an adult malnourished patient cohort with drug abuse and human immunodeficiency virus (HIV) infection with a high mortality rate of 41% [26, 34]. Other studies detected SIADH in 66% of patients with lethal TB meningitis [3] and showed that SIADH was a risk factor for poor outcomes [4]. Adrenal or pituitary insufficiency and brain lesions could cause the severe course of TB meningitis in patients with hyponatremia [12]. Studies highlighted hyponatremia as a marker for overall disease burden [12, 30], as cardiovascular [31, 35], pulmonary [36], and infectious [26, 34] comorbidities and the location of TB [26] are pivotal epidemiologic factors determining disease burden. Conclusively, in patients with verified TB diagnosis, patient history for hyponatremia and current bloodwork should be surveyed to indicate a possible increased mortality risk, and these patients warrant more controls and care.

Hsu et al. established the timeframe of one year to associate TB with episodes of hyponatremia or SIADH. Half of all retrospectively identified initially idiopathic SIADH episodes were associated with pulmonary TB [17], defining one year as diagnostically relevant for follow-up and identification of SIADH cause [17].

The main strength of this analysis was the large cohort obtained at a tertiary care hospital with over 100.000 patients and the long study timeframe for including patients diagnosed with M. tuberculosis infection within the hospital. The main limitation of this paper was the bias brought by clinical decisions in SIADH testing. The usual reasons for applying SIADH tests were symptomatic or more severe hyponatremia or clinical routine such as in-patient treatment. Overall, the hospital tested 7055 of 107.532 patients with hyponatremia. Clinical volemia was assessed indirectly through urine sodium value [15]. However, most endpoints focused on TB-positive patient records, which we thoroughly assessed manually. Hoorn et al. describe SIADH diagnosis as challenging to achieve in clinical praxis, as other types of hyponatremia, such as cerebral salt-wasting syndrome, are difficult to differentiate [13, 15, 37]. Whilst urine sodium value was performed as a surrogate for volume status in this retrospective study, similar to other studies [15], clear demarcation of hyponatremia cause and clinical evaluation of volume status is essential in guiding treatment of TB-associated hyponatremia [13, 14]. Our study included very few patients with both SIADH and TB; further studies exploring this specific subset of patients might be of interest. As our study included only three patients with SIADH and TB, an important limitation is the inability of a more in-depth analysis of this subgroup. In fact, one of the main shortcomings is a lack of pathophysiological explanation, as TB has been reported to both cause SIADH by ectopic production as well as pituitary involvement, and only few studies have analysed the cause of this relevant extrapulmonary effect of TB as classic respiratory infection [4, 8, 11]. Thus, it is essential for further studies to directly elucidate the pathophysiology of TB-associated SIADH.

In-depth analyses of the current study regarding hyponatremia, morbidity and mortality vs location and severity of TB were possibly underpowered.

Our study did not assess hyponatremia correction protocols, as we expected individual clinical application of guideline-conform correction protocols and did not perform a longitudinal analysis of hyponatremia.

## Conclusion

In this study of a large cohort from a tertiary care hospital in a non-endemic area of TB, 0.07% [0.06; 0.09%] of patients presenting with hyponatremia and 0.28% [0.06%; 0.80%] with SIADH, but especially younger patients and patients with high CRP values, were diagnosed with TB. Crucially, patients with moderate to profound hyponatremia had a significantly higher mortality rate than non-hyponatremic patients and thus required heightened medical care.

## Supporting information

S1 Table Database of patients with hyponatremia episodes. Sheet 1 includes data points of patients with hyponatremia in the study. Sheet 2 includes the caption.
(XLSX)

S2 Table Database of patients with tuberculosis. Sheet 1 includes parameters of all patients with tuberculosis, including severity, location, comorbidities and date last seen / death, as well as approximate age, diagnosis date and therapy. Sheet 2 includes the caption.
(XLSX)

S1 Appendix STROBE checklist. The study reporting was performed according to the Strengthening the Reporting of Observational studies in Epidemiology (STROBE) checklist and guidelines.
(PDF)

## Abbreviations

SIADH: syndrome of inappropriate antidiuretic hormone secretion
TB: tuberculosis
ADH: Antidiuretic Hormone
NTM: non-tuberculosis mycobacteria
CRP: C-Reactive Protein
CNS: central nervous system
COPD: chronic obstructive pulmonary disease
HIV: human immunodeficiency virus infection
ICU: intensive care unit
GCS: Glasgow Coma Scale
STROBE: Strengthening the Reporting of Observational studies in Epidemiology
SD: standard deviation
OR: odds ratio
CI: confidence limits
LL: lower limit
UL: upper limit
HR: Hazard ratio
Q1: first quartile
Q3: third quartile
PCR: Polymerase chain reaction
